# Establishment of persistent infection with foot-and-mouth disease virus in BHK-21 cells

**DOI:** 10.1186/1743-422X-8-169

**Published:** 2011-04-14

**Authors:** Xuan Huang, Yong Li, Hui Fang, Congyi Zheng

**Affiliations:** 1 State Key Laboratory of Virology, College of Life Sciences, Wuhan University, Wuhan 430072, China

## Abstract

**Background:**

Foot-and-mouth disease virus (FMDV) is able to cause persistent infection in ruminants besides acute infection and disease. Since the mechanisms of viral persistence and the determining factors are still unknown, *in vitro *systems help explore and reveal mechanisms of persistence *in vivo *by providing useful models for the study of RNA genome mutations and evolution. Ammonium chloride, a lysosomotropic agent that raises intralysosomal pH, reduces the yield of FMDV during infection of BHK-21 cells.

**Results:**

The persistent infection with FMDV serotype O in BHK-21 cells was selected and established readily after treatment of ammonium chloride that acts primarily on the cells. Intact virions were observed located inside the endosomes. Viral genome RNAs and specific proteins were detected in the persistent cells to validate the establishment of viral persistence. Infection of the persistent viruses could not form plaques in host cells but virulence was enhanced. Basing on analysis and comparison of cDNA sequences of resident viruses and wild type viruses, 15 amine acid mutations were found, all of which were located in nonstructural proteins rather than in structural proteins.

**Conclusions:**

Therefore, persistent infection of cell cultures with FMDV is successfully established with some distinctive features. It would be worthwhile to further investigate the mechanisms of viral persistence.

## Background

Virus replication is harmful or pathological for interfering host cell functions [1], but some viruses can reside persistently in their host cells without causing any obvious pathological changes, and keep the way to coevolve [2-5]. Foot-and-mouth disease virus (FMDV), the aetiological agent of foot-and-mouth disease (FMD), which belongs to the *Aphthovirus *genus of the *Picornaviridae *family [6], usually causes an acute, systematic infection of cloven-hooved animals and often produces a persistent noncytocidal infection of ruminants [7]. So far, seven distinct serotypes (A, O, C, Asia1, and South African Territories1, 2, and 3) have been identified with a wide range of subtypes within each serotype [8]. The FMDV genome consists of a single plus-sense stranded RNA of approximately 8.5 kb in length, which contains a single open reading frame flanked by two non-coding regions and a small viral protein VPg linked covalently to the 5'end [9,10]. Replication of FMDV genome RNA occurs via a complementary negative strand RNA. Negative strands were detected in hundredfold lower concentration than positive strands in acutely infected cells, suggesting that each negative strand may serve as a template for the synthesis of many positive strands [11]. Its capsid is composed of 60 copies of each of the four structural proteins VP1-VP4 to form an icosahedral symmetry, in which some viral determinants are involved in the establishment of persistent infection [12].

Among all the picornaviruses, FMDV is the most sensitive to acidic conditions which may in fact enable infection *in vivo *but have a detrimental effect on the virus *in vitro*[13,14]. It has been speculated that the 140S virion of FMDV probably breaks down into 12S pentameric subunits upon entering an acidic endosome, releasing the RNA [10]. The weak lysosomotropic bases that diffuse into acidic endosomes increase the endosomal pH, which result in inhibition of virus infection by human rhinovirus and FMDV [15-17]. Previous studies showed that some lysosomotropic agents that block acidification of endosomes, inhibit FMDV infection, indicating that genome RNA release is dependent on endosomal acidification [13,18,19]. Ammonium chloride, one of the weak lysosomotropic bases that inhibit the pH decrease in endosomes and lysosomes, where the proteolytic processing of viral outer capsid proteins by acid-dependent cellular proteases may occur, can block an early step in infection by intact virions [20-23]. Canning and Fields have demonstrated experimentally that the ammonium chloride can reduce the yield of reovirus and help establish persistent infection rapidly in mouse L cells [24].

To explore and improve our understanding of the mechanisms of foot-and-mouth disease virus persistent infection, a useful model of *in vitro *persistence is needed. De la Torre and his colleagues have established persistent FMDV serotype C (FMDV C) infection by growth of BHK-21(c-13) cells or IBRS-2(c-26) cells that survived standard cytolytic infection with FMDV until massive cell detachment [25,26]. To avoid possible interactions among cells, we selected persistent infection from one cell clone that carried viruses using single cell techniques. Unfortunately, the expected persistence was not observed through several attempts by growth of BHK-21 cells that survived cytolytic infection with FMDV serotype O (FMDV O), the severe serotype that is responsible for outbreaks in large areas of the world. However, the persistent infection with FMDV O in BHK-21 cells can be rapidly selected and established via 20 mM ammonium chloride treatment of infected cells. This *in vitro *model showed that FMDV persistence was established by enhancing the resistance to virus infection at the cellular level facilitated by inhibition of endosome-lysosome system acidification with the aid of ammonium chloride. In addition, we reveal that the persistent viruses possess some distinctive features such as fixed locations, increased virulence, steady replication efficiency in late passages, unimpaired internalization ability, and amino acid replacements in nonstructural proteins.

## Results

### Detection of viral genome RNAs in acutely infected BHK-21 cells maintained in the presence and absence of ammonium chloride

**Figure 1:**
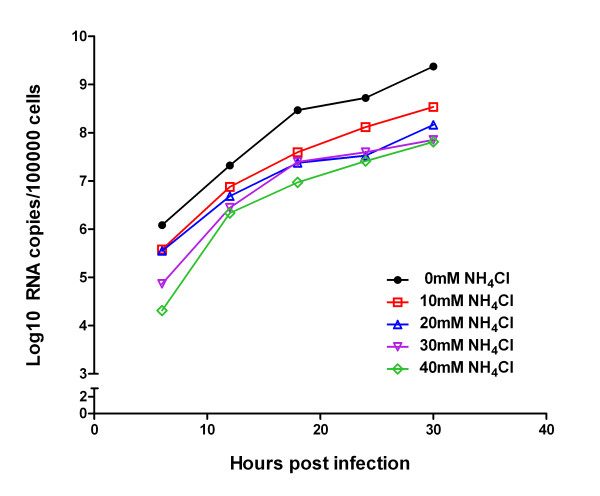
**Effect of ammonium chloride on the yield of FMDV genome RNAs in BHK-21 cells**. 5 × 10^5 ^BHK-21 cells were infected with FMDV at a m.o.i. of 0.01 PFU/cell and maintained in 5% FBS MEM without (0 mM) or with different concentrations of ammonium chloride (10, 20, 30, 40 mM ), respectively. Viral genome RNAs were extracted and performed on qRT-PCR every 6 h from 0 h.p.i. to 30 h.p.i. The unit of Y axis is Log10 RNA copies per 10^5 ^cells, which were evaluated and corrected using qRT-PCR for quantitation of GAPDH. All the cell samples were tested in triplicate.

To investigate the effect of ammonium chloride on the virus yield post-infection, BHK-21 cells in 12-well plates were infected with FMDV and then maintained in 5% FBS MEM supplemented with different concentrations of ammonium chloride. Viral genome RNAs were detected and quantified every 6 h from 0 h post-infection (h.p.i.) till 30 h.p.i. to explore the influences of weak bases on virus infection. The amount of viral RNAs increased sharply and reached a relative peak (exceeding 10^9 ^copies/10^5^cells) at 30 h.p.i. without ammonium chloride. However, it was shown in Figure 1 that the presence of ammonium chloride reduced the accumulations of virus replication by 1-2 magnitudes at all time points detected. Moreover, the extent of suppression on FMDV infection depended on the concentrations of ammonium chloride. The results demonstrated that ammonium chloride can reduce the yield of FMDV during infection of BHK-21 cells.

### Determination of the optimal concentration of ammonium chloride

**Figure 2:**
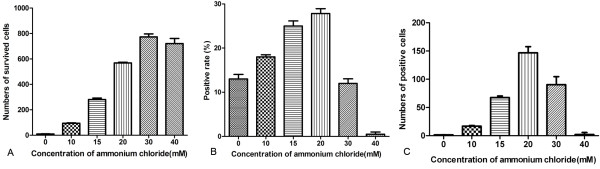
**Treatment of BHK-21 cells with different concentrations of ammonium chloride**. 1 × 10^6 ^BHK-21 cells were infected with FMDV at a m.o.i. of 0.01 PFU/cell and maintained in 5% FBS MEM without (0 mM) or with different concentrations of ammonium chloride (10, 15, 20, 30, 40 mM ) for 48 h, respectively. After removing the alkalescent mediums, infected cells kept in fresh MEM with 10% FBS for 24 h followed by single-cell clone selection and cell counting. (A). The number of cells survived the cytolytic infection maintained in the absence or presence of ammonium chloride. (B). The viral genome RNA positive rates of BHK-21 cells survived. 50 single-cell samples were isolated from each cell suspension survived (300 in total), lysed and run on one-step qRT-PCR assays for detection of viral positive-stranded RNAs. The assay was repeated once. (C). The number of positive cells under the treatment of different concentrations of ammonium chloride.

The viral RNA positive rate (the number of single-cell samples containing viral genome RNAs/the number of single-cell samples tested) and the number of cells survived in FMDV infection maintained in the presence and absence of ammonium chloride were tested and calculated to explore the feasibility of establishing viral persistence with the help of weak bases and the optimal concentration. Confluent BHK-21 cell monolayers (10^6 ^cells) infected with FMDV at m.o.i. of 0.01 PFU/cell resulted in cytopathic effect and massive cell detachment at 48 h.p.i. However, some cells remained attached to the surface, and when provided with fresh medium, they could survive and grow. The number of cells survived (an average value of three independent assays) during FMDV infection maintained in the absence and presence of ammonium chloride (10, 15, 20, 30, 40 mM) differed dramatically (Figure 2A, p < 0.0001). The survival rate reached a peak in the presence of 30 mM ammonium chloride. Meanwhile, the survived cells were rinsed with PBS three times and then trypsinized to yield the cell suspension. In total, 300 single-cell samples were isolated from six different cell suspensions (50 samples per one cell suspension), lysed and then run on one-step qRT-PCR assays[27] to detect the viral genome RNAs (positive-stranded RNAs). The assay was repeated once. The highest positive rate was approximately 25% with the treatment of 20 mM ammonium chloride (Figure 2B). In conclusion, the number of viral RNA positive cells survived was shown in Figure 2C. Approximately 150 viral RNA positive cells out of 10^6 ^cells survived in virus infection maintained in the presence of 20 mM ammonium chloride, a number that is significantly more than when ammonium chloride was absent (only one positive cell out of 10^6 ^cells). Therefore, the establishment of persistent infection with FMDV in BHK-21 cells was feasible and the concentration of 20 mM ammonium chloride was optimal and chosen for selection of viral persistence.

### Selection of persistent infections with FMDV in BHK-21 cells

After 48 h maintenance in 20 mM ammonium chloride, infected cells were allowed to grow in fresh medium for 24 h without the weak base pressure when single cells were isolated and put onto 96-well plates (one cell/well). After 7-12 days incubation at 37°C, single-cell clones were passaged routinely and then viral RNAs were detected using qRT-PCR. 17 positive cell clones were obtained in three independent experiments, named as BHK-Op cells (with a number appended to "Op" indicating passage times, such as BHK-Op15). One of the persistent single-cell clones was selected randomly for virus identifications, while the rest of the positive cell clones were frozen in liquid nitrogen.

### Electron microscopy observations of FMDV particles

**Figure 3:**
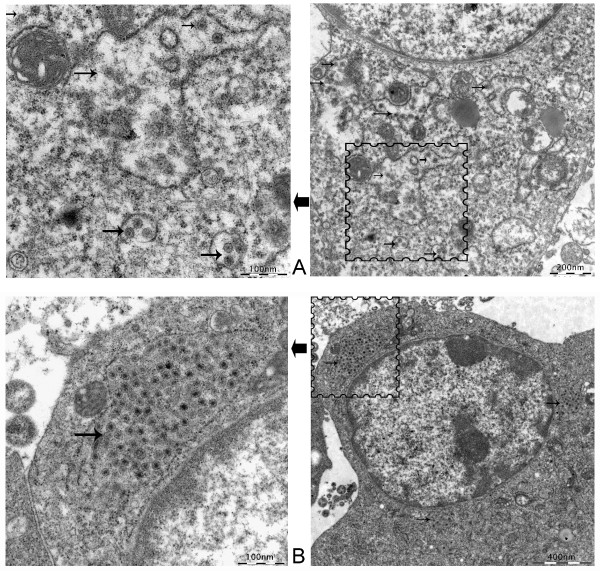
**Electron microscopy (EM) observation of FMDV particles in BHK-Op cells**. A total of 5 × 10^6 ^BHK-Op48 cells and BHK-21 cells infected acutely with FMDV (48 h.p.i.) were collected, fixed and prepared for EM observations. (A). The 48th passage of BHK-Op cells (BHK-Op48). The arrows point out the viral particles. (B). BHK-21 cells infected acutely with FMDV. The arrow marks the viral particles.

BHK-Op48 cells were selected randomly for electron microscopy assays to confirm the establishment of persistent infection. FMDV particles were observed successfully in BHK-Op cells, which dispersed in the cytoplasm and were located in some small endosomes with a diameter about 25 nm (Figure 3A). There were less virions observed in the persistent cells compared to the acutely infected cells (Figure 3B) and locations of viral particles differed in the two cells. As shown in Figure 3B, virions in acutely infected cells were located mainly in endosomes close to the nucleus in large quantities. However, virions in persistent cells were located in some endosomes that dispersed in the cytoplasm in very small quantities (not more than 10, usually 2 or 3). The results suggested that ammonium chloride may play an important role in the establishment of persistent infection in our assays for ammonium chloride helped prevent the virions releasing after entering endosomes and reduced the yield of viruses via raising the pH of endocytic organelles.

### Morphological features of BHK-Op cells

**Figure 4:**
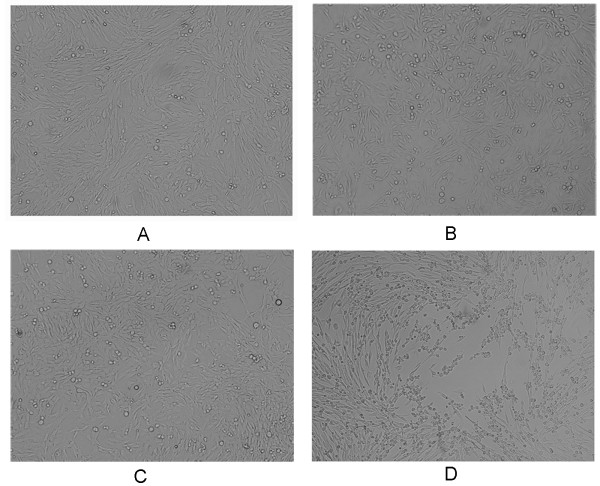
**Morphological observation of BHK-Op cells**. (A). BHK-21cells. (B). BHK-Op8 cells. (C). BHK-Op48 cells. (D). BHK-21 cells acutely infected with FMDV.

Compared to normal BHK-21 cells (Figure 4A), there is not any visible change in the morphological appearances of BHK-Op cells both in early stages (BHK-Op8, Figure 4B) and late stages (BHK-Op48, Figure 4C). Although BHK-Op cells coexisted with viruses, they did not manifest cytopathic effects which were observed in acutely infected cells (Figure 4D), suggesting that the persistence of FMDV does not change morphological features of BHK-21 cells and has no influence on the cell growth and proliferation.

### Detection of viral proteins

**Figure 5:**
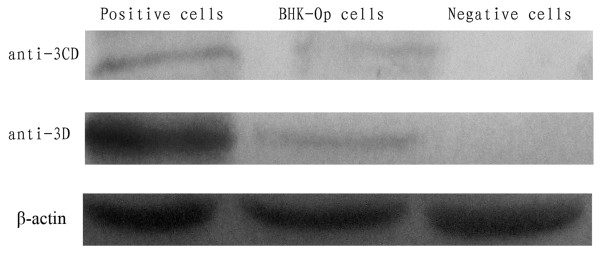
**Western blot analysis for detection of viral proteins 3D and 3CD**. Western blot analysis detected FMDV specific proteins 3D (viral RNA dependent RNA polymerase) and 3CD (a protein precursor consisting of two different proteins 3C and 3D) in BHK-Op48 cells and BHK-21 cells infected acutely (30 h.p.i., positive control). β-actin was included as control of protein loading and the number of cells.

Western blot analysis detected FMDV specific proteins 3D (viral RNA dependent RNA polymerase) and 3CD (a protein precursor consisting of two different proteins 3C and 3D) in BHK-Op48 cells and acutely infected BHK-21 cells (30 h.p.i., positive control) (Figure 5). The results showed that the amounts of 3D and 3CD proteins in the persistent cells were less than that of the proteins in positive cells.

### Quantitation of FMDV positive-stranded and negative-stranded RNAs

**Figure 6:**
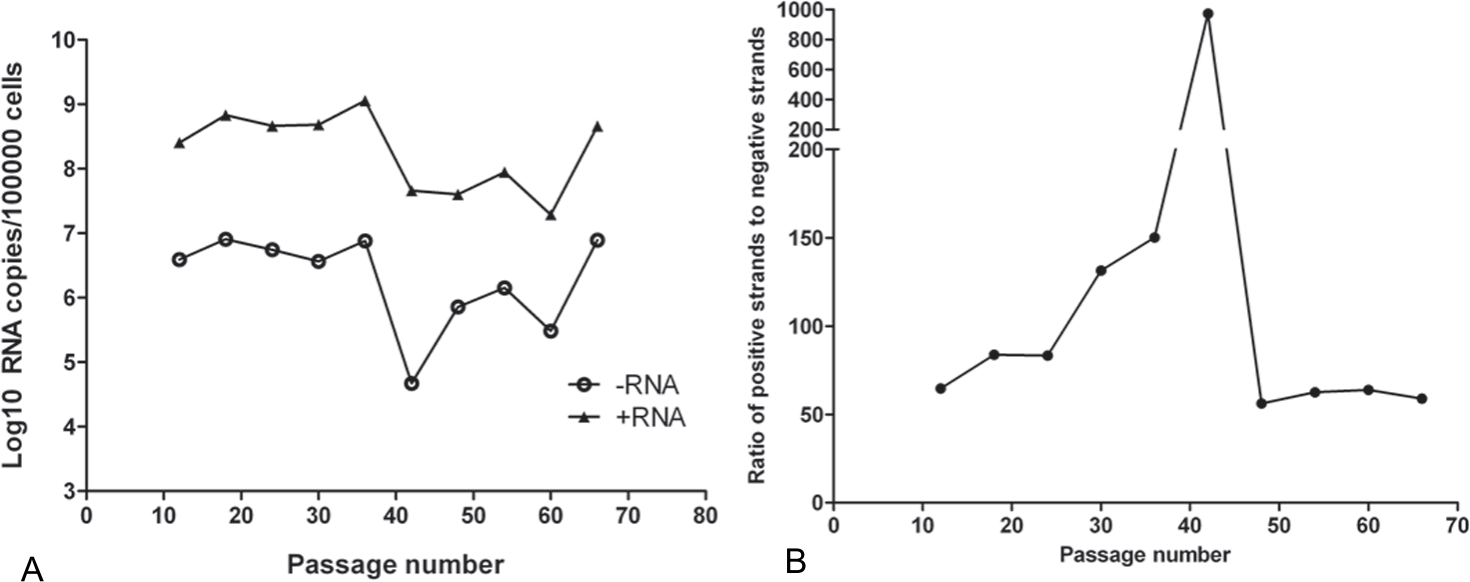
**The amounts of viral RNAs and the levels of viral RNA replication dynamics in different passages of BHK-Op cells**. Viral RNAs of BHK-Op12, 18, 24, 30, 36, 42, 48, 54, 60, 66 were extracted to perform on duplex qRT-PCRs. (A) Quantitation of viral positive strands and negative strands. (B). The ratios of positive-stranded RNAs to negative-stranded RNAs.

Viral RNAs of BHK-Op cells (BHK-Op12, 18, 24, 30, 36, 42, 48, 54, 60, 66) were extracted to perform on the duplex qRT-PCR assays [28]. The results shown in Figure 6A indicated that the amount of FMDV positive strands ranged from 10^7 ^to 10^9 ^copies per 100000 cells (the number of cells was evaluated and corrected using qRT-PCR for quantitation of house-keeping gene GAPDH, data not shown), with a relative peak at passage of 36. The ratios of positive strands to negative strands increased gradually in the early stages and reached a climax of 973 at BHK-Op42, and then traced back to low values in the late stages (Figure 6B), suggesting that viruses and host cells might be struggling for a balance in order to coexist before passage 36. It was speculated that the viruses and host cells were struggling for balance in early passages and the persistent viruses lived harmoniously with host cells with steady levels of viral replication in late passages after a rapid decrease of the viral loads.

### Detection of cell viability in the presence of ammonium chloride

**Figure 7:**
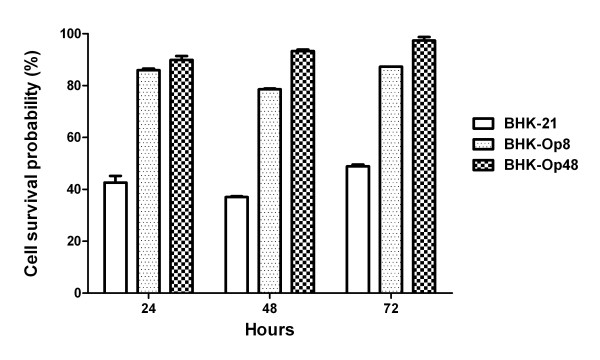
**Cell viability of BHK-21 cells and BHK-Op cells in the presence or absence of 20 mM ammonium chloride**. MTT assays were used to determine the viability of BHK-21 cells and persistent BHK-Op cells treated with 0 mM (negative control for this assay) or 20 mM ammonium chloride. Cell survival rate was measured at three different time points: 24 h, 48 h maintenance in ammonium chloride and 48 h maintenance in ammonium chloride then in fresh 10% FBS MEM for 24 h. The results are detected from two independent experiments.

To determine whether the cells were affected first by ammonium chloride, MTT assays were carried out to show resistance of cells to the weak bases. As shown in Figure 7, when treated with 20 mM ammonium chloride for 24 h and 48 h, the survival probabilities of BHK-21 cells were not more than 45%, indicating that the presence of ammonium chloride had a serious influence on cell growth. However, the survival probabilities of BHK-Op cells exceeded 75% which were much higher than that of BHK-21 cells (p < 0.001). In conclusion, the persistent cells which were selected and obtained with the help of ammonium chloride were more resistant to ammonium chloride compared with normal cells, implying that ammonium chloride acted on the host cell. Furthermore, the facts that the survival rates of late passages of BHK-Op cells (BHK-Op48) were much higher than that of early passages of BHK-Op cells (BHK-Op8) indicated that the effects of ammonium chloride on the host cell retained and may be enhanced through cell passage.

### The influences of ammonium chloride on the growth of persistent viruses

BHK-Op8 cells, BHK-Op48 cells and acutely infected BHK-21 cells were frozen at -80°C for at least 1 h, and then thawed at 37°C, respectively. Freezing and thawing were repeated for a total of three cycles for lysis of cells and release of viruses. After 30 min of centrifuge at 3000 g, 4°C, the supernatants were filter-sterilized with 0.22 μm-filter and collected as virus suspensions (The viruses in BHK-Op8 cells, BHK-Op48 cells and acutely infected BHK-21 cells were named as FMDV-Op8, FMDV-Op48 and FMDV wild type.). The virus suspensions were performed on RNA extraction using Trizol LS reagent according to the manufacturer's instructions and then run on qRT-PCR for quantitation of viral RNAs.

Confluent BHK-21 cells in 12-well plates were infected with FMDV wt, FMDV-Op8 and FMDV-Op48 at equal amounts of viral RNAs. After a 1 h absorption period, the inoculum was removed and 1 ml fresh medium was added (with or without 20 mM ammonium chloride). To address the influences of ammonium chloride on the growth of persistent viruses, cell samples were collected every 6 h from 0 h post-infection (h.p.i.) till 30 h.p.i. and then total RNAs were extracted and quantified.

**Figure 8:**
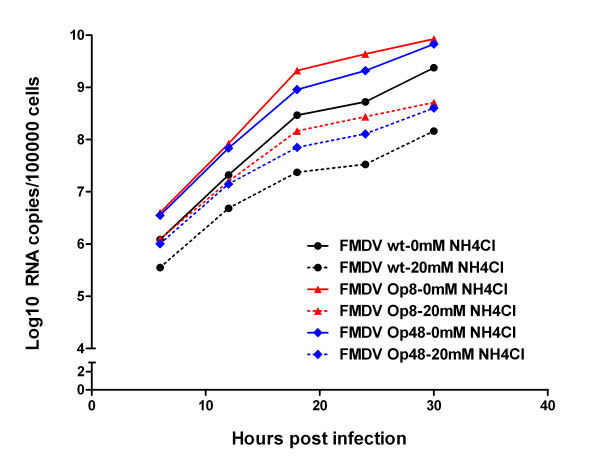
**Growth of FMDV wt and FMDV-Ops in BHK-21 cells maintained in the presence and absence of 20 mM ammonium chloride**. Confluent BHK-21 cells in 12-well plates were infected with FMDV wt, FMDV-Op8 and FMDV-Op48 at equal amounts of viral RNAs and then maintained with or without 20 mM ammonium chloride. Cell samples were collected every 6 h from 0 h post-infection (h.p.i.) till 30 h.p.i. and then total RNAs were extracted and quantified via qRT-PCR. All the cell samples were tested in triplicate.

As shown in Figure 8, the presence of 20 mM ammonium chloride reduced the yield of FMDV wt at all time points detected. Likewise, the viral RNAs in BHK-21 cells infected with FMDV-Ops incubated with 20 mM ammonium chloride were 10-fold less than that of cells maintained free of ammonium chloride. The inhibition rates of virus yield in wild type viruses and persistent viruses were similar. In conclusion, infection of wt viruses and persistent viruses were both sensitive to ammonium chloride treatment. In other words, FMDV-Ops did not gain the resistance to ammonium chloride.

### Infectivity and virulence of FMDV-Ops

**Figure 9:**
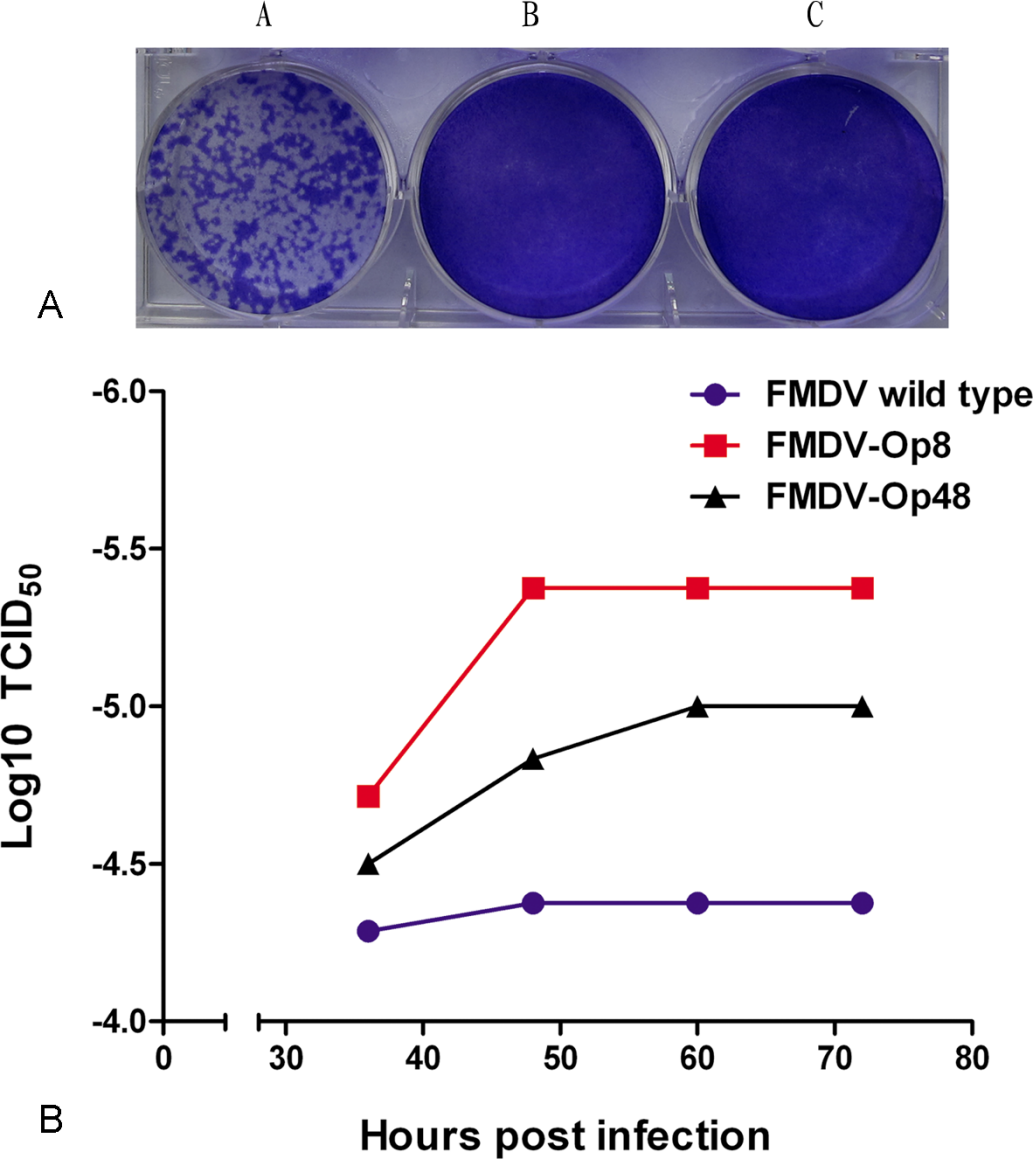
**Infectivity and virulence assays of FMDV-Ops and wt**. (A) Plaque assays. Plaque assays were designed to compare the infectivity of FMDV-Ops with wild type. Confluent BHK-21 cells in 6-well plates were infected with FMDV wt, FMDV-Op8 and FMDV-Op48 at equal amounts of viral RNAs and were overlaid with 3 ml maintenance medium (2% FBS) containing 0.8% purified agarose and incubated for 3d. Plaques were stained with 1 ml 1% crystal violet in 10% paraformaldehyde. A. FMDV wt. B. FMDV-Op8. C. FMDV-Op48. (B) TCID_50 _assays. TCID_50 _assays were designed to compare the virulence of FMDV-Ops with wild type. The TCID_50 _value of FMDV-Ops and wt were calculated every 12 h from 36 h.p.i. till 72 h.p.i.

Plaque assays and TCID_50 _assays were designed to compare the infectivity and virulence of FMDV-Ops with FMDV wt. As mentioned in the text, suspensions of FMDV wt, FMDV-Op8 and FMDV-Op48 were filter-sterilized and quantified using qRT-PCR. Both plaque assays and TCID_50 _assays were carried out using equal amount of viral genome RNAs of the three viruses for virus infection. The TCID_50 _value of the three viruses were calculated every 12 h from 36 h.p.i. Neither FMDV-Op8 nor FMDV-Op48 infection can form plaques in BHK-21 cells after 3 d maintenance in the semi-solid mediums while infection of FMDV wild type (FMDV wt) resulted in plenty of large plaques under the same condition (Figure 9A). However, the fact that TCID_50 _of FMDV-Op8 (10^-5.375^) and FMDV-Op48 (10^-5^) were smaller than that of FMDV-wt (10^-4.375^) at 72 h.p.i. implied that the virulence of FMDV-Ops was enhanced compared to that of wild type viruses (Figure 9B) although the persistent viruses lost the ability to form plaques.

### Virus internalization

**Figure 10:**
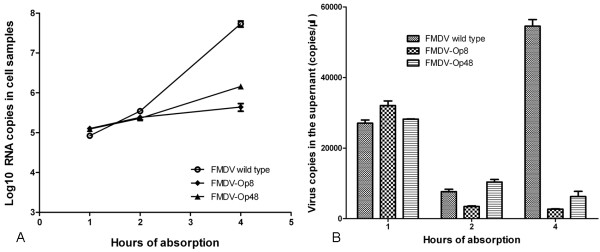
**The internalization assays of FMDV-Op8, FMDV-Op48 and FMDV wt**. (A) Quantitation of cell samples after 1 h, 2 h and 4 h absorption of three viruses. (B) Quantitation of the supernatants from each cell sample. All the cell samples and supernatants were tested in triplicate.

BHK-21 cells were infected with FMDV-Op8, FMDV-Op48 and FMDV wt at equal amount of viral genome RNAs with different absorption periods (1 h, 2 h and 4 h). Both the supernatants and cell samples were collected and run on qRT-PCR for detection of viral RNAs. The results in Figure 10A showed that the amounts of viral RNAs in FMDV wt infected cells and FMDV-Op infected cells were similar to each other after 1 h (2 h) absorption whereas the amounts of viral RNAs in FMDV wt infected cells were higher than 10-fold of that in FMDV-Op infected cells at 4 h.p.i. (after 4 h absorption), indicating that all the three viruses could bind to the cell surfaces and only the wild type viruses began to replicate within 4 h.p.i. Moreover, the FMDV wt rather than FMDV-Ops released viruses to the supernatant at 4 h.p.i.(Figure 10B) which was consistent with the results in Figure 10A.

### Mutations in the genome of FMDV-Op

To evaluate the spectrum of mutations in the persistence of FMDV, we analyzed and compared the sequences of full cDNA clone of FMDV wt and FMDV-Op48. Protein sequence comparison and analysis of FMDV-wt and FMDV-Op48 revealed that all the amine acid mutations were located in nonstructural proteins rather than structural proteins (Table 1). No mutations were found in structural proteins, which was consistent with the results of virus internalization assays, indicating that the recognition of cell receptors of the persistent viruses were not influenced and the persistent viruses can bind to the cell surfaces and enter into the host cells as wild type viruses do.

**Table 1:** The amino acid substitutions in the persistent virus*

Viral protein	Mutation types and positions
2C	Glu→ Ala (aa 1174)

2C	Val→ Leu (aa 1389)

2C	Pro→ Thr (aa 1394)

2C	Asn→ Ser (aa 1418)

3A	Ile→ Val (aa 1498)

3A	Thr→ Asn (aa 1522)

3A	Lys→ Val (aa 1535)

3A	Asp→ Asn (aa 1563)

3B1	Leu→ Arg (aa 1584)

3B3	Lys→ Asn (aa 1642)

3C	Glu→ Gln (aa 1698)

3C	Asp→ Asn (aa 1753)

3C	Gly→ Arg (aa 1779)

3D	Glu→ Lys (aa 2009)

3D	Val→ Leu (aa 2187)

* The nucleotide sequence of wild type virus (serotype O, Akesu/58/2002) was used as reference, GenBank accession no. AF511039.

## Discussion

Foot-and-mouth disease is one of the most highly contagious diseases, which has been deemed as the most important constraint to international trade in animals and animal products [10]. Its causative agent, FMDV, is able to cause persistent infection in ruminants in addition to causing acute infection and disease, which makes control efforts even more costly. Since the mechanisms of viral persistence and the determining factors are still unknown, *in vitro *systems (viral persistence in cultured cells) may help explore and reveal mechanisms of persistence *in vivo *by providing useful models for the study of RNA genome mutations and evolution. De la Torre had successfully established persistent FMDV C infection by growth of BHK-21(c-13) that survived standard cytolytic infection until massive cell detachment [25].

The lysosomotropic weak bases, which diffuse into acidic endosomes and raise the pH of endocytic organelles, prevent the required low pH-dependent proteins conformational changes leading to virus genome release. As expected for a virus with an endosomal entry pathway (such as FMDV), ammonium chloride neutralized the acidic endolysosome compartments, blocking an early step in virus infection. Previous studies have confirmed that ammonium chloride can reduce the yield of reovirus after infection and help establish persistent infection rapidly in mouse L cells [24]. So far as we know, ammonium chloride has not been used for selection and establishment of FMDV persistence previously except studies in our lab. The influences of ammonium chloride on the growth of FMDV were investigated first to analyze the feasibility of establishing viral persistence with the help of weak bases. The results in Figure 1 demonstrated that the presence of ammonium chloride (10-40 mM) can reduce the yield of virus replication at all time points detected.

A newly developed single-cell qRT-PCR [27] was used in our assay to seek the optimal concentration of ammonium chloride used in selection of persistent infection with FMDV serotype O. The viral RNA positive rate and the number of cells survived determine the probability of selection of persistent infection. In conclusion, approximately 0.015% (150/10^6^) of the cells may be virus persistent cell clones treated with 20 mM ammonium chloride, which is 100-fold larger than that of cells in the absence of ammonium chloride (0.00013%). The results explained exactly why the persistence of FMDV O (Akesu/58/2002) was not established merely by growth of BHK-21 cells survived. The effect of the multiplicity of infection on the establishment of persistent infections with poliovirus in Hep-2 cell cultures had been previously shown that low multiplicity of infection favored establishment of persistent infection [29]. Based on these results, a very low multiplicity of 0.01 PFU/cell was used for infection to establish FMDV persistence in the assay.

Evidence that BHK-Op cells were persistently infected with FMDV includes (1): Detection of FMDV genome RNAs by qRT-PCR in different passages of BHK-Op cells. (2): Detection of FMDV specific proteins 3D and 3CD. The 3D and 3CD proteins were detected positive in BHK-Op48 cells and the amounts of the viral proteins were less than that in acutely infected cells. (3): Observation of intact virions. The FMDV particles were successfully observed in BHK-Op48 cells under the transmission electron microscope.

In order to facilitate establishment of persistent infection, ammonium chloride may act primarily on the host cell or on the virus itself. To make it clear, we explored the effects of ammonium chloride on both persistent cells and viruses. As shown in Figure 8, infections of wt viruses and persistent viruses were both sensitive to ammonium chloride treatment. On the other hand, results of MTT assays showed that the survival probabilities of BHK-Op cells were much higher than that of BHK-21 cells maintained in 20 mM ammonium chloride. In other words, the persistent cells acquired resistance to ammonium chloride. Interestingly, after ammonium chloride treatment virus-resistant cells and virus-sensitive cells were also selected and obtained. In conclusion, ammonium chloride exerted its primary effect on the host cell in the process of viral persistence establishment. It is easy to explain the emergence of virus-resistant cells because ammonium chloride may result in phenotypic protection of certain subpopulations of cells by preventing the virions from releasing, which was consistent with previous studies [24,25]. However, virus-sensitive cells selected during the establishment of persistence have never been reported yet, which needs further and deeper research.

The virus suspensions of FMDV-Op8, FMDV-Op48 and FMDV wt were quantified by qRT-PCR to adjust and equalize the amount of viruses used for infection in the plaque assays and TCID_50 _assays. The fact that infections of both FMDV-Op viruses resulted in no plaques in BHK-21 cells while FMDV wt infection formed many clear and visible plaques indicates that the infectivity of FMDV-Op may be influenced and impaired in the process of viral persistence. However, the result of TCID_50 _assays implies that the virulence of FMDV-Op viruses was enhanced compared to that of wild type viruses. Therefore, virus internalization assays were designed and carried out to investigate whether the bindings of viruses to cell surfaces were blocked and the possible reasons for vanishing plaques. After a 1 h (2 h) absorption period, viral genome RNAs were detected in cells infected with FMDV-Op8, FMDV-Op48 and FMDV wt respectively, demonstrating that all the three viruses could bind to cell surfaces and access to the host cells. The amounts of viral RNAs in FMDV wt infected cells were quantified more than 10-fold of that in FMDV-Op infected cells at 4 h.p.i. Therefore, it appears that FMDV-wt went through a natural infectious cycle in the host cells with active viral proteins transcription and genome replication. Meanwhile, the life cycles of FMDV-Ops may be retarded and most of the viral particles may lose the ability to uncoat and retain in the endosomes, which is likely to be one of the reasons for lost plaques. Furthermore, it has been proved that ammonium chloride inhibits the required low pH-dependent proteins conformational changes, and blocks viral penetration of the cytoplasm rather than inhibiting viral entry by blocking uptake at the cell surface [15,18,30], which helps to clarify the function and mechanism of ammonium chloride treatment.

Protein sequence analysis of wt viruses and persistent viruses revealed that all the amine acid mutations were situated in functional proteins rather than capsid proteins. Based on the genome comparisons of 103 isolates of FMDV representing all seven serotypes [31], 6 out of 15 mutations were the mutations of invariant amino acids (1 in 2C protein, 1 in 3A protein, 3 in 3C protein and 1 in 3D protein.) which may have some effect on the functions of the viral proteins. Viral 3C protein is related to the trypsin family of serine proteases and cleaves most of the proteins from polyproteins. Mutations in the conservative regions of 3C protein may result in the decline of activity. Nayak and his coworkers found that the R95 and R97 residues of 3C protein were crucial for RNA binding in virus replication [32]. It was found that the R95S and R97S mutant transcripts were non-infectious; no plaques were observed at 8 h post electroporation. However, the influences on virus life cycle of the amino acid mutations are unclear and further investigations are needed. Microarray experiments are underway to attempt to search host cell proteins or factors pertaining to FMDV persistence in BHK-21 cells.

## Conclusions

In summary, persistent infection with FMDV O (Akesu/58/2002) was established successfully with the aid of ammonium chloride which can not be selected and established merely by growth of BHK-21 cells survived cytolytic infection. We found there were some unique features of the persistent cells. First, the virions in persistent cells were observed located mainly in endosomes scattered in the cytoplasm. Secondly, infection of the persistent viruses can not form plaques in host cells but the virulence of FMDV-Ops was enhanced compared to that of wild type viruses. Thirdly, there were no mutations of amino acid in structural proteins which is not in line with the previous studies [12]. Moreover, the virus internalization assays confirmed that the FMDV-Ops can bind to cell surfaces after 1 h absorption. We believe that this newly established persistent cell culture can provide a system for elucidating mechanisms of viral persistence *in vivo*. Besides, *in vitro *persistence is also a useful tool for investigating virus and cell evolution and facilitating the study of virus-receptor interactions, which can be served to define genetic determinants of virulence and to identify viral and host cell determinants involved in the establishment of persistent infection. Moreover, the establishment of persistent infections in cells *in vitro *under the treatment of ammonium chloride may have important implications for the use of lysosomotropic drugs *in vivo*.

## Methods

### Cell and virus

BHK-21 cell is a clone of cells provided by China Center for Type Culture Collection (CCTCC) where this study was conducted. The virus strain of serotype O FMDV (Akesu/58/2002) used in the present study was derived from the Lanzhou Veterinary Research Institute, Chinese Academy of Agriculture Sciences, which was cloned by three successive isolations of plaques formed on the BHK-21 cells. BHK-21 cells were cultured in Minimum Essential Medium (MEM, Life Technologies, Carlsbad, U.S.A.) supplemented with 10% heat-inactivated fetal bovine serum (FBS, Life Technologies, Carlsbad, U.S.A.) and penicillin (100 units/ml)-streptomycin (0.1 mg/ml) at 37°C with 5% CO_2 _and used to propagate virus stocks and measure virus titers in plaque assays.

### RNA extraction

BHK-21 cells were centrifuged and homogenized with 400 μl Trizol reagent (Life Technologies, Carlsbad, U.S.A.) in 1.5 ml Eppendorf polypropylene tubes while 250 μl liquid samples were homogenized in 750 μl Trizol LS reagent (Life Technologies, Carlsbad, U.S.A.). Total RNA was extracted according to the manufacturer's instructions.

### One-step quantitative RT-PCR (qRT-PCR) assay for detection of viral genome RNAs in acutely infected BHK-21 cells

qRT-PCR was performed using the Platinum^® ^Quantitative RT-PCR ThermoScript™ one-step Mastermix Reagents Kit (Life Technologies, Carlsbad, U.S.A.). The PCR primers and probe (listed in Table 2) for detecting FMDV RNA located within the viral 3D genes encoding RNA-dependent RNA polymerase, which are based on nucleotide sequencing of serotype O (Akesu/58/2002, GenBank accession no. AF511039; [27]). The qRT-PCR was performed in a final volume of 50 μl consisting of 3 mM MgSO_4_, 0.2 mM of each dNTP, 1 μl ThermoScript™ Plus/Platinum^® ^*Taq *Enzyme Mix, 40 U RNase inhibitor, 300 nM probe, 500 nM forward primer and reverse primer, 5 μg bovine serum albumin (New England Bio Labs, Beverly, U.S.A.) and 10 μl RNA samples or standard RNA samples. The standard RNA templates were prepared as described elsewhere [27]. The RT-PCR was carried out on Bio-Rad CFX96 real-time PCR detection system (Bio-Rad Laboratories, Berkeley, U.S.A.). Based on the manufacturer's protocol, cDNA was synthesized at 50°C for 30 min, and the PCR profile was 95°C for 5 min, followed by 40 cycles of 94°C for 30 s and 60°C for 90 s. All the cell samples were tested in triplicate. Data analyses were carried out using Bio-Rad CFX manager.

### Establishment of persistent infection

#### Virus infections and treatment of infected cells with ammonium chloride

BHK-21 cells in 6-well plates (or 12-well plates) were infected with FMDV at a multiplicity of infection (m.o.i.) of 0.01 PFU/cell. After 1 h of absorption at 37°C, cells were washed for 1 min with 0.1 M phosphate buffer (pH 6.0) to inactivate unabsorbed virions, and washed again extensively with MEM. The infection was allowed to proceed in MEM (5% FBS) supplemented without or with 10, 15, 20, 30, 40 mM ammonium chloride for 48 h, respectively. After removing the mediums with ammonium chloride, infected cells were washed extensively with MEM and then kept in fresh MEM supplemented with 10% FBS for 24 h followed by single-cell clone selection [27].

### One-step single cell qRT-PCR assay

Single cells were isolated and lysed as described previously [27]. Single cell qRT-PCR was performed using the same protocol described above. The PCR profile was 95°C for 5 min, followed by 45 cycles of 94°C for 30 s and 60°C for 90 s. Data analyses were carried out using Bio-Rad CFX manager.

### Selection of cell clones infected persistently with FMDV

After 48 h treatment of ammonium chloride and 24 h maintenance in fresh MEM, infected cells were rinsed with PBS three times and then trypsinized at 37°C for 5 min. 9 ml 10% FBS MEM was then added to end trypsinization. Single cells were isolated using a micromanipulator (Narishige, Tokyo, Japan) and put onto a 96-well plate (one cell per well) containing fresh MEM with 10% FBS [27]. The isolated single cells in separate wells were allowed to form a monolayer, passaged and frozen as usual which have been described previously [25].

### Electron microscopy

A total of 5 × 10^6 ^BHK-21 cells, BHK-Op48 cells and acutely infected BHK-21 cells (48 h.p.i.) were centrifuged and washed with PBS (0.1 M) twice, respectively. Harvested cells were fixed by 2.5% glutaraldehyde at 4°C overnight. Samples were postfixed in 1% osmium tetroxide for 2 h at room temperature, rinsed, and then dehydrated in an up-graded ethanol series (30%, 50%, 70%, 80%, 90%, 95%, and 100%) and embedded in epoxy resin, and ultrathin sections were double stained in uranyl acetate and lead citrate. The ultrathinsections (60-80 nm) were observed under a FEI TECNAI G^2 ^transmission electron microscope (FEI, U.S.A.).

### Western-blotting analysis

Viral specific proteins, 3D (viral RNA dependent RNA polymerase) and 3CD were identified by western blot following the procedures: proteins extracted from persistently and acutely infected BHK-21 cells by RIPA buffer (20 mM Tris-HCl pH 7.5, 100 mM NaCl, 0.5% NP-40, 0.5 mM EDTA, 0.5 mM PMSF, 0.5% protease inhibitor cocktail [Roche]) were subjected to 12% sodium dodecyl sulphate (SDS)-polyacrylamide gel, electrotransferred onto a polyvinylidene fluoride (PVDF) membrane, blocked with 5% nonfat milk in PBS, and reacted with rabbit anti-FMDV 3D serum as primary antibodies. Alkaline phosphatase (ALP)-conjugated goat anti-rabbit immunoglobulin G antibody (Sigma, U.S.A.) was used as the secondary antibody. After washing with TBS three times, membrane-bound antibodies were detected with Nitro Blue etrazolium/5-bromo-4-chloroindol-2-yl phosphate.

### Quantitation of FMDV positive- and negative-stranded RNAs by duplex qRT-PCR

The duplex qRT-PCR for simultaneous detection of FMDV positive-stranded RNAs and negative-stranded RNAs has been developed and evaluated in our lab [28]. On the basis of the characteristics of FMDV genome, 2B primers and probe were used for detection of positive-stranded RNAs while 3D primers-probe mentioned above were designed for quantitation of negative-stranded RNAs aiming at synthesis of different DNA fragments (or non-overlapped fragments) in the reverse transcription step. The sequences of the primers and probes used in the duplex qRT-PCR were shown in Table 2.

**Table 2:** Synthetic oligonucleotides used for qRT-PCR

Primers/probes	Oligonucleotide sequences(5'-3')	Nucleotide positions	Gene	Fragment length
FP	ACAAAACACGGACCCGACTT	3977-3996	2B	67 bp

RT	CTTTTACTCCTATGGCCAGTTCCT	4020-4043		

Probe	**HEX**-AACCGCCTGGTGTCCGCGTTT-**BHQ1**	3998-4018		

				

FP	GAACACATTCTTTACACCAGGAT	7184-7206	3D	121 bp

RT	CATATCTTTGCCAATCAACATCAG	7281-7304		

Probe	**FAM**-ACAACCTACCGCCGAGCCAATTC-**TAMRA**	7254-7276		

				

FP	AAGGCCATCACCATCTTCCA	127-147	GAPDH	87 bp

RT	GCCAGTAGACTCCACAACATAC	192-213		

Probe	**FAM-**AGCGAGATCCCACCAACATCAAATGGG**-BHQ1**	149-175		

Reverse transcription was performed in a 20 μl reaction mix containing 0.5 μl Transcriptor Reverse Transcriptase (20 U), 4 μl 5 × Transcriptor RT Reaction Buffer (Roche Diagnostics, Berlin, Germany), 0.5 μl RNase inhibitor (20 U, TAKARA BIO, Shiga, Japan), 2 μl 10 mM dNTP mix, 2 μl 25 μM 2B RT primer and 2 μl 25 μM 3D FP primer, 4 μl DEPC water and 5 μl standard templates or samples. The initial denaturation at 65°C for 5 min was done with RNA samples and primers followed by snap cooling on ice. Then, after adding enzyme, buffer and dNTPs, the cDNA synthesis was carried out at 55°C for 30 min followed by heating at 85°C for 5 min to inactivate transcriptase. cDNA products were cooled on ice and stored at -30°C until use.

The optimum PCR reaction mixture contained 5 μl of each 10-fold dilution of the 2B and 3D standard cDNAs used to generate standard curves or 5 μl cDNA of cell samples, 5 μl of 10 × LA PCR Buffer||, 500 nM each of forward and reverse primers for 2B and 3D, 200 nM 3D probe, 400 nM 2B probe, 400 nM of each dNTP and 2.5 U LA Taq. Distilled water was added to a total volume of 50 μl. Amplification and detection were performed with a Bio-Rad CFX96 real-time PCR detection system under the following conditions: 95°C for 3.5 min, followed by 40 cycles of 94°C for 30 s and 60°C for 90 s. All the cell samples were tested in triplicate.

The house-keeping gene glyceraldehyde-3-phosphate dehydrogenase (GAPDH) was examined and quantified as an internal control for FMDV quantitation. Specific primers and probe (see in Table 2) for GAPDH (GenBank accession no. DQ403055) were designed using Primer Express 2.0 (Applied Biosystems, Foster City, U.S.A.). GAPDH mRNAs were detected with both one-step and routine two-step qRT-PCRs using the same protocols and conditions depicted above.

### MTT assays

MTT (3-(4,5-Dimethylthiazol-2-yl)-2,5-diphenyltetrazolium bromide, a yellow tetrazole), is reduced to purple formazan in living cells. MTT assays were used to determine the viability of BHK-21 cells and persistent BHK-Op cells treated with 0 mM (negative control) or 20 mM ammonium chloride. Approximately 5000 cells (BHK-21 cells, BHK-Op8 cells and BHK-Op48 cells) were plated separately into each well of 96-well plates in a 100 μl volume and incubated overnight at 37°C with 5% CO_2_, and then the supernatants were replaced by 100 μl 5% FBS MEM containing 0 mM or 20 mM ammonium chloride. Cell viability was measured at three different time points: 24 h, 48 h maintenance in ammonium chloride and 48 h maintenance in ammonium chloride then in fresh 10% FBS MEM for 24 h. 20 μl 5 mg/ml MTT (Sigma, St. Louis, U.S.A.) were added to each well and cells were incubated in the culture hood for 4 h. After that, the supernatants were removed and 100 μl DMSO were added to dissolve the MTT (formazan). Cell viability was determined with a microplate reader at 490 nm. Each experiment was performed in sixteen replicate wells for each sample and was repeated once.

### Plaque assays and TCID_50 _assays

Virus was titered by standard plaque assay [33] with some modifications. Briefly, confluent monolayer of BHK-21 cells in 6-well plates as well as serial 10-fold dilutions of wild type viruses and persistent viruses with equal viral RNA copies in MEM were prepared, 0.1 ml virus suspension was added to each well in duplicate for each dilution after removal of the medium and washing thrice. Infected cultures were then incubated at 37°C for 60 min with gentle shaking every 15 min to assure uniform adsorption. Following this, cell monolayers were overlaid with 3 ml maintenance medium (2% FBS) containing 0.8% purified agarose and incubated at 37°C in a 5% CO_2 _incubator until plaques formed. 3-4 days later, plaques were stained using 1 ml 1% crystal violet in 10% paraformaldehyde and recorded after washing the monolayer with tap water gently. TCID_50 _assays were carried out using the routine method described previously [34].

### Statistical analysis

The significance of the results was tested using the unpaired t test and the one-way ANOVA analysis of variance using commercially available software (Graphpad Prism, San Diego, USA). A P value less than 0.05 was considered significant. All values reported in the text and figures were expressed as means ± standard error of mean.

## Competing interests

The authors declare that they have no competing interests.

## Authors' contributions

HX and LY conceived the study and designed the experiments. HX and FH carried out the experimental work. HX, LY and ZCY wrote the paper. All authors have read and approved the final manuscript.
